# 

**Published:** 1893-08

**Authors:** 


### CONTRIBUTORS TO VOLUME XXXIII.

| | |
|---|---|
| Abbott, Frank W., M. D. | Buffalo. |
| Angell, Edward B., M. D. | Rochester. |
| Bartlett, Frederic W., M. D. | Buffalo. |
| Burr, C. B., M. D. | Pontiac. |
| Cary, Charles, M. D. | Buffalo. |
| Clark, Edward, M. D. | Buffalo. |
| Clark, Horace, M. D. | Buffalo. |
| Clausius, M. F., M. D. | Winnebago. |
| Cott, George F., M. D. | Buffalo. |
| Crockett, M. A., M. D. | Buffalo. |
| Daggett, Byron H., M. D. | Buffalo. |
| Doyle, Gregory, M. D. | Syracuse. |
| Fritz, William C., M. D. | Buffalo. |
| Gram, Franklin C., M. D. | Buffalo. |
| Hartwig, Marcell, M. D. | Buffalo. |
| Haynes, Francis L., M. D. | Los Angeles. |
| Himmelsbach, G. A., M. D. | Buffalo. |
| Hopkins, Henry Reed, M. D. | Buffalo. |
| Howe, Lucien, M. D. | Buffalo. |
| Hubbell, Alvin A., M. D. | Buffalo. |
| Hurd, A. W., M. D. | Buffalo. |
| Hulbert, George F., M. D. | St. Louis. |
| Ingraham, Henry D., M. D. | Buffalo. |
| Jones, Allen A., M. D. | Buffalo. |
| Judson, A. B., M. D. | New York. |
| King, Clarence, M. D. | Machias. |
| Kingsley, Mrs. Carlton A. | Westfield. |
| Krauss, William C., M. D. | Buffalo. |
| Long, Benj. G., M. D. | Buffalo. |
| Long, J. W., M. D. | Richmond. |
| Lothrop, Thomas, M. D. | Buffalo. |
| Love, Isaac N., M. D. | St. Louis. |
| Manley, Thomas H., M. D. | New York. |
| Mann, Matthew D., M. D. | Buffalo. |
| Miller, John A., Ph. D. | Buffalo. |
| Milliken, Samuel E., M. D. | New York. |
| Mynter, Herman, M. D. | Buffalo. |
| Parmenter, John, M. D. | Buffalo. |
| Potter, William Warren, M. D. | Buffalo. |
| Putnam, James Wright, M. D. | Buffalo. |
| Renner, W. Scott, M. D. | Buffalo. |
| Robinson, F. Byron, M. D. | Chicago. |
| Rochester, DeLancey, M. D. | Buffalo. |
| Sellstedt, L. G., Esq. | Buffalo. |
| Starr, Elmer, M. D. | Buffalo. |
| Tait, Lawson, Esq., M. D. | Birmingham, Eng. |
| Thompson, J. C., M. D. | Buffalo. |
| Thornbury, Frank J., M. D. | Buffalo. |
| Weigel, Louis A., M. D. | Rochester. |
| Wende, G. W., M. D. | Buffalo. |
| Wheeler, William A., M. D. | New York. |
| Whipple, Electa B., M. D. | Buffalo. |

Buffalo Academy of Medicine.
Philadelphia County Medical Society.
Medical Society of the County of Erie.

### ILLUSTRATIONS TO VOLUME XXXIII.

PAGE.

CONCERNING POSTURE. (B. H. DAGGETT, M. D.)
Fig. 1.—Dorsal Posture—Knees and thighs flexed . . . . . . . . 86
Fig. 2.—Knee,-thigh,-chest Posture . . . . . . . . . . . . . . . . 88

RADICAL CURE OF INGUINAL HERNIA. (S. E. MILLIKEN, M. D.)
Fig. 1.—Dissection of Sac and Exposure of Cord . . . . . . . . 147
Fig. 2.—Suturing of Conjoined Tendon and Poupart's Ligament . 148
Fig. 3.—Suturing of Flaps of External Oblique . . . . . . . . . 149

HYDRO-NEPHROSIS FROM VALVULAR STRICTURE OF URETER. (HERMAN MYNTER, M. D.)
Fig. 1.—Incision through Valvular Stricture and Sac . . . . . . 222
Fig. 2.—Appearance After Operation . . . . . . . . . . . . . . . 222

LARYNGEAL TUBERCULOSIS. (HORACE CLARK, M. D.)
View of Larynx in a Typical Case . . . . . . . . . . . . . . . . 276

TRICUSPID INSUFFICIENCY. (F. J. THORNBURY, M. D.)
Anadicrotic Wave Synchronous with Auricular Systole . . . . . 280

A NEW METHOD OF ARTIFICIAL RESPIRATION IN ASPHYXIA NEONATORUM. (J. HARVIE DEW, M. D.)
Fig. 1.—Opening of the Epiglottis by Gravity . . . . . . . . . . 666
Fig. 2.—Depression of the Pelvis and Lower Extremities . . . . . 667
Fig. 3.—Forcible Expiration by Arching the Lumbar Region Backward and Bending the Child Upon Itself . . . . . . . . . . . 668
Fig. 4.—Expulsion of Mucus by Elevating Buttocks and Depressing Head . . . . . . . . . . . . . . . . . . . . . . . . . . 672

HEGAR'S SIGN OF PREGNANCY. (J. W. LONG, M. D.)
Fig. 1.—Intra-vaginal Finger in Anterior Vaginal Fornix—Abdominal Hand Behind Fundus . . . . . . . . . . . . . . . . . . . . . 706
Fig. 2.—Intra-vaginal Finger Behind Cervix, Abdominal Hand Between Symphysis and Fundus . . . . . . . . . . . . . . . . . 707
Fig 3.—Hand Between Symphysis and Fundus, Finger in Rectum . . 707

BLADDER GYMNASTICS AND AUTO-IRRIGATION. (B. H. DAGGETT, M. D.)
Fig. 1.—The Daggett Canula . . . . . . . . . . . . . . . . . . . 714
Fig. 2.—Posture for Auto-irrigation of the Bladder . . . . . . . . 715

PORTRAIT OF HENRY VAN AERNAM, M. D., facing . . . . . . . . . . 739

### Society Meetings.

AMERICAN ELECTRO-THERAPEUTIC ASSOCIATION.—The third annual meeting of the American Electro-Therapeutic Association will be held in Chicago, September 12th, 13th, and 14th, at Appollo hall, Central Music Hall Block. Members of the medical profession interested in electro-therapeutics are cordially invited to attend.

AUGUSTIN H. GOELET, M. D., *President.*
MARGARET A. CLEAVES, M. D., *Secretary.*

---

CIVIL SERVICE EXAMINATION FOR JUNIOR ASSISTANT PHYSICIANS.—An open competitive examination of candidates for the position of Junior Assistant Physician in the State Hospital service will be held at the rooms of the State Civil Service Commission, in the Capitol, Albany, on Tuesday, September 5, 1893, at 10 A. M. An applicant must be a graduate of a legally chartered medical college, and have had at least one year's actual experience on the medical staff of a public general hospital. For application blanks, address Clarence B. Angle, Secretary New York Civil Service Commission, Albany, N. Y.

THOMAS CARMODY,
ALBANY, N. Y., July 24, 1893. *Chief Examiner.*

### Literary Notes.

HERNIA, Its Radical and Tentative Treatment in Infants, Children, and Adults. By Thomas H. Manley, A. M., M. D., Visiting Surgeon to Harlem Hospital ; Consulting Surgeon to the Fordham Hospital, etc., etc. This work is illustrated by sixty-five engravings and drawings, with a full history of the ancient and modern operations for the hernial infirmity of every type, in both sexes, along with a full description of the varied anatomical types of the condition and the multiplicity of technique of modern times. It also embraces an entire chapter on Cocaine Analgesia, as a substitute for Pulmonary Anesthesia, with a full and complete set of rules for its indications and technique. Price, $3 ; mailed to any address. Published by the Medical Press Co., Limited, 1725 Arch street, Philadelphia, to which all orders should be addressed.

---

PHYSICAL EDUCATION for June (published at Springfield, Mass. ; $1 per year), is a number of unusual interest. An article on The Gymnastic Treatment of the Feeble Minded shows how, through the connection of exercise of muscle, it is possible to exercise and thus develop those parts of the brain that have to do with muscular contraction, and that thus, as well as in other ways described, the brain can be stimulated to develop. That this is not mere theory, is shown by the fact that Dr. Gulick, the writer, is describing simply what he has himself done, giving the reasons for it.

A continued article, by Dr. Hitchcock, of Cornell, and R. F. Nelligan, of Amherst, is on Wrestling, and consists mainly of illustrations taken from life, showing the various holds and breaks in the catch-as-catch-can style.

Besides these and major articles, there is much of minor interest.

---

THE *Cosmopolitan* offers $1,500 in four prizes, of $1,000, $300, $100, and $100, respectively, for the four water colors which shall be chosen by a committee from such drawings as may be submitted by the artists of the United States or Europe, on or before twelve o'clock on the 1st day or December, 1893. The subjects are to be selected from the life of Christ, taking those scenes which teach in the highest forms the lessons of love, patience, humility, and forbearance, with fidelity, as far as may be, to the actual surroundings and conditions of the period. The treatment should be calculated for single-page reproduction in *The Cosmopolitan*, in size five by eight inches. The subjects to be suitable, as far as possible, for use in stained glass for church or cathedral. The originals for which prizes are awarded will become the property of *The Cosmopolitan.* The drawings should be shipped, securely packed, and addressed: "Submitted to Art Committee, *Cosmopolitan Magazine*, Sixth avenue and Eleventh street, New York," and in the upper left-hand corner: "Not to be opened before the 1st day of December, 1893."

---

A LITERARY SENSATION.—"Uncle Tom's Cabin" has certainly "broke loose"! The copyright of this most famous of American novels, by Mrs. Stowe, has recently expired, which frees its publication from the monopoly of the high-priced publishers, and though in anticipation of this fact they have within a few months greatly reduced its price, now that it is really "unchained," the consequences are something surprising. John B. Alden, publisher, of New York, issues several editions, selling them only *direct* (not through agents or booksellers); one in good type, paper covers, for 5 cents, sent post-paid, or the same bound in cloth for 10 cents, with postage 7 cents extra; also an excellent large-type edition, on fine paper, handsomely bound in cloth for the price of 25 cents, postage 10 cents. Surely a copy of "Uncle Tom's Cabin" will soon be found in every home where it is not already. Mr. Alden sends a thirty-two page pamphlet describing many of his publications free, a catalogue of 128 pages of choice books, a veritable "literary gold mine" for book-lovers, for 2 cents. Address John B. Alden, publisher, 57 Rose street, New York.

Notice to Contributors.—We are glad to receive contributions from every one who knows anything of interest to the profession. Articles designed for publication in the Journal should be handed in before the first day of the month. The Editors are not responsible for the views or opinions of contributors. All communications should be addressed to the Managing Editor, 284 Franklin St., Buffalo, N. Y.

